# Visual-manual response selection produces dual-task interference in auditory-verbal memory encoding

**DOI:** 10.1007/s00426-026-02273-x

**Published:** 2026-03-31

**Authors:** Sandra Hensen, Iring Koch, Patricia Hirsch

**Affiliations:** Institute of Psychology, RWTH Aachen University, Jaegerstrasse 17/19, 52066 Aachen, Germany

## Abstract

In the present study, we investigated how response selection in a visual-manual reaction-time (RT) task affects encoding in an auditory-verbal free recall memory task. In dual-task conditions, the visual stimulus was presented first, followed by the auditory memory item after either a short stimulus-onset asynchrony (SOA) of 300 ms or a long SOA of 700 ms. After the set of memory items was encoded, participants verbally recalled the to-be-remembered items after performing a short distractor activity. The memory task and RT task were also performed in single-task conditions to assess global dual-task costs. Results showed decreased recall accuracy in the memory task in a dual-task condition compared to a single-task condition, reflecting dual-task costs. Moreover, we observed worse memory performance with short SOA compared to long SOA. In addition, in dual-task conditions we analyzed on a trial-level whether a processing conflict in the RT task (i.e., congruency effect) modulated recall accuracy in the memory task. Results showed that recall accuracy was worse for items encoded during incongruent trials of the RT task compared to congruent trials. Together, the findings indicate that memory for auditory items suffers under dual-task demands with visual-manual response selection. In the RT task, we also observed dual-task costs, with longer RT with short SOA compared to long SOA. Overall, the observed dual-task interference can be interpreted in terms of a shared limited capacity between memory encoding and response selection, but also the roles of attentional prioritization, preparatory effects and overlapping verbal codes are discussed.

## Public significance statement

The present study shows that when people make quick decisions in visual-manual tasks while trying to memorize spoken information into long-term memory, their memory performance declines. It is suggested that decision-making and encoding information into memory interfere with each other and possible explanations like shared limited cognitive resources, attentional prioritization or preparatory effects are discussed. Overall, our findings help to explain why performing two tasks at once can lead to information loss in memory, especially in conflict-driven situations while decision-making.

## Introduction

The impact of multitasking on memory performance has been studied for several decades. It has been shown that dual-task requirements during *memory encoding* usually lead to a decline in later recognition and recall accuracy (e.g., Baddeley et al., 1984; Craik et al., 2018; Craik et al., 1996; Fernandes & Moscovitch, 2000; Naveh-Benjamin et al., 2000). Studies that introduced dual-task demands during *memory retrieval* also showed negative effects on recall accuracy (e.g., Lozito & Mulligan, 2006; Rohrer & Pashler, 2003), but also recall latency (e.g., Carrier & Pashler, 1995). However, regarding recall accuracy, most studies observed larger dual-task costs at the stage of memory encoding compared to memory retrieval (see e.g., Naveh-Benjamin et al., 2006).

A common task to investigate dual-task effects in memory is the free-recall memory task. In this task, participants are asked to encode visual or auditory word lists. After a short distractor activity, the encoded items need to be retrieved verbally or by typing. Dual-task situations can be implemented either during the encoding phase or the retrieval phase of the memory task. Typically, in those dual-task situations, a discrete reaction-time (RT) task (i.e., a task that requires responses after each stimulus) is performed simultaneously during encoding or retrieving the memory items. The RT task and memory task are also performed in isolation, in so-called single-task conditions. Typically, performance is worse in dual-task conditions compared to single-task conditions (i.e., dual-task costs; see Fischer & Janczyk, 2022, for a review). Even though many studies found dual-task costs in the memory task and RT task at the memory encoding phase of a memory task, the underlying cognitive mechanisms are still not fully understood (see Naveh-Benjamin et al., 2014). In the present study, we aim to close this research gap and investigate the impact of visual-manual response selection on auditory-verbal memory encoding using a Psychological Refractory Period (PRP)-like paradigm.

### PRP paradigm with two discrete RT tasks

The PRP paradigm is typically used to investigate dual-task interference between discrete RT tasks. In each trial, participants perform two RT tasks with varying degrees of temporal overlap, defined by the stimulus-onset asynchrony (SOA), which is the time interval between the stimulus presentation for Task 1 and that for Task 2 (e.g., Janczyk & Kunde, 2020; Pashler, 1994, for reviews). A common finding is that RT to the second stimulus increases as the SOA decreases, an effect referred to as the PRP effect (e.g., Hirsch et al., 2018; Koch et al., 2018; Pashler, 1994; Welford, 1952).

Models explaining dual-task interference with two discrete RT tasks typically assume that the processing of each task is subdivided into perceptual processing, response selection, and response execution. The PRP effect is typically hypothesized to arise from capacity limitations at the “central” stage of response selection (Pashler, 1994; see e.g., Hirsch & Koch, 2024; Koch et al., 2018, for more recent reviews). This limitation could be, for example, due to a “central” all-or-none bottleneck that assumes that processing in Task 2 cannot begin until processing in Task 1 has ended (e.g., the “Central Bottleneck” model; Pashler, 1984, 1994). Another explanation arises from the idea of capacity sharing, which suggests capacity-limited processing at the “central” stage, resulting in a slowdown in Task 1 and Task 2 during the time when capacity is shared (i.e., task performance gets worse the more two processes that require “central” capacity overlap in time; e.g., the “Central Capacity Sharing” (CSS) model; Tombu & Jolicoeur, 2003). Besides capacity limitations at the “central” stage, also other explanations were suggested as reasons for observed dual-task costs, like interference due to shared similarities between tasks (e.g., both tasks require the same verbal or spatial central code, Wickens, 2008; or working memory (WM) subsystem, Baddeley & Hitch, 1994) or a lack of preparation for processes in the tasks (e.g., less preparation is possible for Task 2 when Task 1 needs to be prepared; De Jong & Sweet, 1994; see also Lyphout-Spitz et al., 2024). However, these models were designed to account for dual-task interference in two RT tasks. The present study focuses on the influence of an RT task on a subsequent memory task, requiring encoding of information for later free recall.

### PRP-like paradigm with a memory task and discrete RT task

Studies investigating memory effects in dual-task situations also used PRP-like paradigms, but instead of two discrete RT tasks, they combined memory tasks with discrete RT tasks (Jolicoeur 1999a, b; Jolicoeur and Dell’Acqua 1998; Koch and Jolicoeur 2007; Koch et al. 2003; Koch and Prinz 2002, 2005). For example, Jolicoeur (1999a) investigated whether an auditory speeded pitch discrimination task (Task 1) and a visual memory encoding task (Task 2) create dual-task interference. In Experiment 1 and 2, participants were presented with an auditory target (tone), and after varying SOAs (50, 150, 250 or 600 ms) a visual target (one or two letters) was presented. The difference between the experiments was that the visual target was immediately masked (letters replaced with “$” signs) in Experiment 2, whereas in Experiment 1 no mask was used. Participants were instructed to make a speeded pitch discrimination for the presented auditory target and to encode the visual target, which needed to be recalled after the response to the auditory target (i.e., a short-term memory task). Results showed that recall for the visual targets (Task 2) decreased with short SOA only when a mask was used. In Experiment 2, accuracy in the auditory task (Task 1) was also significantly lower with the short SOA compared to the longer SOAs (RTs were unaffected).

Based on these findings, Jolicoeur (1999a) concluded that encoding information into short-term memory (i.e., short-term consolidation; STC) also involves “central” processes. With shorter SOA, response selection to the auditory target was not finished when the visual memory target was encoded, resulting in a greater temporal overlap between these processes compared to trials with longer SOA. Due to the temporal overlap of “central” processes, STC was slowed down or postponed, resulting in a decay of the unconsolidated information (see also Jolicoeur & Dell’Acqua, 1998). Further, Jolicoeur (1999a) stated that masking of the visual target immediately was important to cut short the sensory input and control for visual persistence, as otherwise Task 2 performance might not be sensitive to dual-task interference from Task 1 due to the possibly that the visual perceptual representation outlasts until processing in Task 1 was completed.

### Impact of process interference on memory encoding

The observed interference between STC and response selection raises the question of whether memory encoding is also influenced by processing conflicts at response selection. Such processing conflicts can be induced in response-conflict tasks (e.g., Stroop task or Eriksen task). In response-conflict tasks, stimuli contain both task-relevant and task-irrelevant features. A trial is congruent when the task-relevant and the task-irrelevant features are associated with the same response, and a trial is incongruent when these features are linked to different responses. RT and error rates are typically higher in incongruent trials than in congruent trials, resulting in a congruency effect (Botvinick et al., 2001; see Lu & Proctor, 1995, for a review). Memory performance might be worse when a memory item is encoded during an incongruent trial compared to a congruent trial, because participants possibly focus on conflict resolution in the conflict task (as Task 1) and neglect the encoding of the memory item^1^. Another possible explanation could be that due to the prolonged response selection process in incongruent trials compared to congruent trials, “central” processes in both tasks could have a larger temporal overlap (i.e., the same logic as with short SOA and long SOA).

In a previous study, we examined the impact of task-specific processing conflicts in a visual-manual spatial Stroop task on auditory-verbal memory encoding (Hensen et al., 2024, Experiment 2). Participants were asked to auditorily encode memory items (words) in either single-task conditions or dual-task conditions (i.e., simultaneously with the spatial Stroop task). In a spatial Stroop task, the spatial meaning of a location word was classified. Location words appeared at different positions on the screen (e.g., the location word “left” could appear either on the left or right side of the screen). The spatial meaning of the location word and its spatial location on the screen could either match or mismatch, resulting in congruent or incongruent trials. Presentation of the memory items and the Stroop stimuli was time-locked to examine the effect of the processing conflict on a trial-level in the spatial Stroop task on recall performance in the memory task. After participants encoded all memory items and performed a distractor activity, the memory items were verbally recalled in a retrieval phase (i.e., delayed recall). For this long-term memory task, results showed decreased recall performance in dual-task conditions compared to single-task conditions, but there was no significant impact of the processing conflict in the spatial Stroop task on recall performance. As a manipulation check, for the spatial Stroop task, we found the standard congruency effect. We also tested the Stroop task in single-task conditions and found worse performance in dual-task conditions, hence mutual dual-task interference across both the Stroop task and the long-term memory task. We concluded that dual-task interference between the long-term memory task and the spatial Stroop task is primarily influenced by general capacity-sharing and not further influenced by task-specific processing conflicts.

However, another explanation for why the effect of processing conflict on memory performance was not significant in our previous study could be that some participants prioritized the encoding of the memory item over response selection in the Stroop task. In the experiment, we did not explicitly emphasize which task should be performed first (i.e., participants were instructed to respond as fast as possible to the Stroop task while still ensuring to encode the memory item carefully). Further, the visual Stroop stimuli were presented on the screen for a long duration (i.e., 2000 ms). Thus, it might be the case that for some participants recall performance was not sensitive for the manipulation of the processing conflict as response selection was postponed. The present study further investigated this possibility.

### Present study

The aim of the present study was to investigate the impact of visual-manual response selection on auditory-verbal memory encoding in a delayed free recall memory task. More specifically, we were interested in the impact of processing conflicts during response selection (i.e., congruent vs. incongruent trials) on recall performance of simultaneously encoded memory items. To this end, we used a PRP-like paradigm, with a strictly defined Task 1 and Task 2. Further, we presented the visual stimuli only briefly to prevent that response selection processes are postponed in time (e.g., Jolicoeur 1999a). In addition, we added an SOA manipulation to provide further insights into the cause of interference between memory and response selection processes. The memory task and spatial Stroop task used were identical to those in our previous study (Hensen et al., 2024), but the overall onset and duration of stimulus presentation changed. Both tasks were performed in single-task conditions and dual-task conditions at the memory encoding phase of the memory task to explore global dual-task costs.

For the long-term memory task (i.e., our Task 2), we hypothesized that recall accuracy would be worse in a dual-task condition, when combined with a Stroop task (Task 1) compared to a single-task condition. Moreover, we expected decreased recall accuracy with short compared to long SOA (i.e., PRP-like effect). This is a novel aspect of our study as we are not aware of existing studies examining SOA effects on memory encoding in a long-term memory requirement. We also expected worse recall accuracy in the memory task (Task 2) when memory items are encoded during an incongruent trial of the spatial Stroop task (Task 1) compared to during a congruent trial. Again, such a finding would be novel given the lack of existing research using the PRP methodology in the context of a Task 2 that is essentially a long-term memory task (but requires memory encoding and thus updating of the already encoded item list in each individual trial).

For the spatial Stroop task (Task 1), we hypothesized to find a standard congruency effect for RT and error rates, which represents a basic manipulation check to be able to interpret corresponding effects in the memory task (Task 2), but we also expected increased RT and error rates in a dual-task condition compared to a single task condition, confirming our previous study. We did not formulate strong predictions for the effect of SOA on performance in the spatial Stroop task (Task 1) or any interaction effects, as the focus of the present study is on memory performance in Task 2.

## Method

### Apparatus, stimuli and responses

For the visual stimuli, the German location words “LINKS”, “RECHTS”, “OBEN”, and “UNTEN” (left, right, up, and down) were used. Moreover, a fixation cross (+), random generated three-digit numbers, and the German word “FEHLER” (error) were visually presented. All visual stimuli were displayed in white on a black background in the font “Arial”. The height of all visual stimuli was 4 cm, but with different widths (e.g., the fixation cross was 4 × 4 cm). Except for the location words, all stimuli were presented in the screen center. The location words “LINKS” and “RECHTS” were presented either on the left or right side of the screen, with 4 cm to the screen center. The location words “OBEN” and “UNTEN” were displayed either 4 cm above or below the screen center. The positioning of all location words was counterbalanced, resulting in 50% congruent and 50% incongruent trials. The screen was a 24-inch screen, and the viewing distance was approximately 70 cm.

For the auditory stimuli, 154 German, psychology-related three-syllable words were used. The words were mainly selected from the Diagnostic and Statistical Manual of Mental Disorders (American Psychiatric Association, 2013; e.g. “Depression”, “Emotion”, “Gedächtnis”, “Entwicklung”, “Kognitiv”, “Gewohnheit”, “Kontrolle”; see Table 1 in the Appendix for a full list of all words). To create the audio files, a text-to-speech converter with a female voice was used. All audio files were set to a duration of one second. To generate the word lists, each audio file was randomly assigned to one list. In total, two lists with five words and nine lists with 16 words were created with a randomized word order for each participant. Further, a 600 Hz “beep” sound was used as a signal tone for the beginning of the retrieval phase. The auditory stimuli were presented binaurally via headphones.

Manual responses were detected with a QWERTZ keyboard, by pressing the keys “Y” for the response left, “X” for the response right, arrow key “up” for the response up, and “down” for the response down. The keys were pressed with the middle and index fingers of the left and right hand. All vocal responses were recorded with a standalone microphone. PsychoPy 3 (Peirce et al., 2019) was used to run the experiment.

### Procedure

After filling out an informed consent and data protection sheet as well as a demographic questionnaire, participants received instructions in both verbal and written form. They started with a practice phase of the experimental tasks. The main experiment included single-task conditions for the spatial Stroop task and memory task and two different dual-task conditions with either a short or long SOA. After the experimental blocks, participants received a debriefing. The total time of the experiment was approximately 45 min.

In the single-task spatial Stroop condition, participants had to classify the spatial meaning of a location word while ignoring its spatial location on the screen. Each trial started with the presentation of a location word on the screen for 300 ms. After this time, the location word disappeared, and a fixation cross appeared in the screen center for a fixed duration of 1200 ms. Within these 1500 ms, manual responses were possible. In case of a correct response, the fixation cross persisted for another 1000 ms until the start of the next trial. In case of an error or timeout (no response within 1500 ms), visual error feedback was displayed for 300 ms, followed by a fixation cross for 700 ms. (i.e., the total duration of one trial was 2500 ms). Participants completed 16 trials per block with a randomized stimulus presentation, but the appearance of each location word was counterbalanced (i.e., each location word appeared equally often, resulting in 8 congruent and 8 incongruent trials per block).

In the single-task memory condition, participants performed an encoding phase, followed by a distractor activity and retrieval phase. In the 40-second encoding phase, a fixation cross was visually displayed, and 16 words were auditorily presented at a 2.5 s rate. After all words were encoded, a randomly generated three-digit number appeared on the screen and participants counted backwards by threes for 30 s. A signal tone cued the start of the 60-second retrieval phase, in which participants recalled the previously encoded words as fast as possible in any order.

In the dual-task conditions, the spatial Stroop task (Task 1) was performed during the memory encoding phase of the memory task (Task 2). Each trial started with the visual presentation of a location word for 300 ms. The auditory presentation of a word from the word list was either after a short SOA of 300 ms or a long SOA of 700 ms (blocked design). In both conditions, the location word disappeared after 300 ms, and a fixation cross was presented in the screen center for 1200 ms. Within these first 1500 ms, manual responses were possible. In case of a correct response, the fixation cross persisted for another 1000 ms. In case of an error or timeout, the error feedback was displayed for 300 ms. followed by a fixation cross for 700 ms. After all 16 trials were completed, the distractor activity and retrieval phase were performed like in the single-task memory condition.

The experiment started with three different practice blocks, beginning with a practice block of the spatial Stroop task (8 trials). After that, a practice block of the memory task was performed with a 5-element word list, followed by a 15-second distractor activity and a 20-second retrieval phase. Lastly, a practice block for the dual-task condition (with a 5-element word list and 5 trials of the spatial Stroop task) was conducted before the experimental blocks started. The main experiment included three blocks for each of the four conditions (single-task spatial Stroop, single-task memory, dual-task with short SOA, and dual-task with long SOA), resulting in 12 experimental blocks which were counterbalanced with a Latin square design. Moreover, it was counterbalanced across participants which wordlists were used in either the single-task condition or dual-task conditions.

### Design

For conceptual reasons, we divided the single-task condition and the dual-task conditions with short and long SOA into two non-orthogonal contrasts. First, to examine global dual-task costs, we compared single-task performance with that in dual-task conditions with long SOA. Second, we examined the effect of dual-task specific temporal overlap by comparing dual-task performance with long SOA (low dual-task overlap) with that with short SOA (high temporal overlap). We decided to compare only the dual-task condition with long SOA (excluding the dual-task condition with short SOA) with the single-task condition, to not combine two heterogenous conditions (i.e., short SOA and long SOA) into a single factor level and to reduce redundancy with the dual-task specific analysis. As the dual-task condition with long SOA is considered the “easier” of the two dual-task conditions, this analysis was sufficient to investigate whether performing two tasks simultaneously would result in costs compared to performing the tasks completely isolated from each other.

*Task 1*. For the spatial Stroop task, a 2 × 2 repeated-measurements design was used with the independent within-subject variables task load (single-task vs. dual-task at memory encoding with long SOA) and congruency (congruent vs. incongruent) to analyze general dual-task costs.

To analyze dual-task specific effects of temporal task overlap, we used a 2 × 2 repeated-measurements design (single-task trials excluded) with the independent within-subject variables SOA (short vs. long) and congruency (congruent vs. incongruent). The dependent variables were RT and error rate.

*Task 2*. For the memory task, we also specified two analyses. To assess global dual-task costs, we again compared single-task and dual-task performance using task load (single-task vs. dual-task at memory encoding with long SOA).

Further, we used a 2 × 2 repeated-measurements design (single-task trials excluded) to analyze the effect of SOA (short vs. long) and congruency (congruent vs. incongruent trial in the spatial Stroop task). The dependent variable was recall total (i.e., the percentage of words correctly recalled from the most current list).

### Participants

Seventy-two psychology students (55 female; mean age = 21.22 years, *SD* = 1.95) took part in the experiment (additional *n* = 7 participants were tested but due to the exclusion criteria excluded from all analyses, see results section). Participants were native German speakers and reported normal or corrected-to-normal vision and hearing acuity. All gave informed consent for participation and received partial course credit or monetary compensation (10€ per hour) in exchange.

An a priori sample size calculation was conducted using MorePower 6.0 (Campbell & Thompson, 2012). The sample size was estimated for an analysis of variance (ANOVA) with the most complex design; two within-subject factors with each two levels. As effects of interest, all main effects were selected (main effects of task load and congruency; SOA and congruency) based on our hypothesized effects. With a medium to large effect size (ηp² = 0.11; based on results of the previous study, Hensen et al., 2024) and an alpha of 0.05, the analysis showed that at least 66 participants were required to achieve a power of 0.8. Due to counterbalancing, 72 participants were recruited (i.e., we were able to detect effect sizes of ηp² = 0.10).

## Results

Data was analyzed with frequentists analysis, using IBM SPSS. All analyses were calculated at α = 0.05. For all analyses, the practice blocks were discarded. Further, for RT analysis of the spatial Stroop task, trials with an error (10.3%) and trials deviating more than +/- 3 standard deviations from each participant’s individual mean RT per condition (0.3%) were eliminated. Participant’s data sets were excluded from the analysis if the error rate exceeded 40% in one or more block(s) of the spatial Stroop task or if zero words were recalled in one or more block(s) of the memory task (*n* = 7). We report the results separately for the spatial Stroop task (Task 1) and the memory task (Task 2).

### Spatial Stroop task (Task 1)

#### Single-task condition vs. dual-task condition with long SOA

**Fig. 1:**
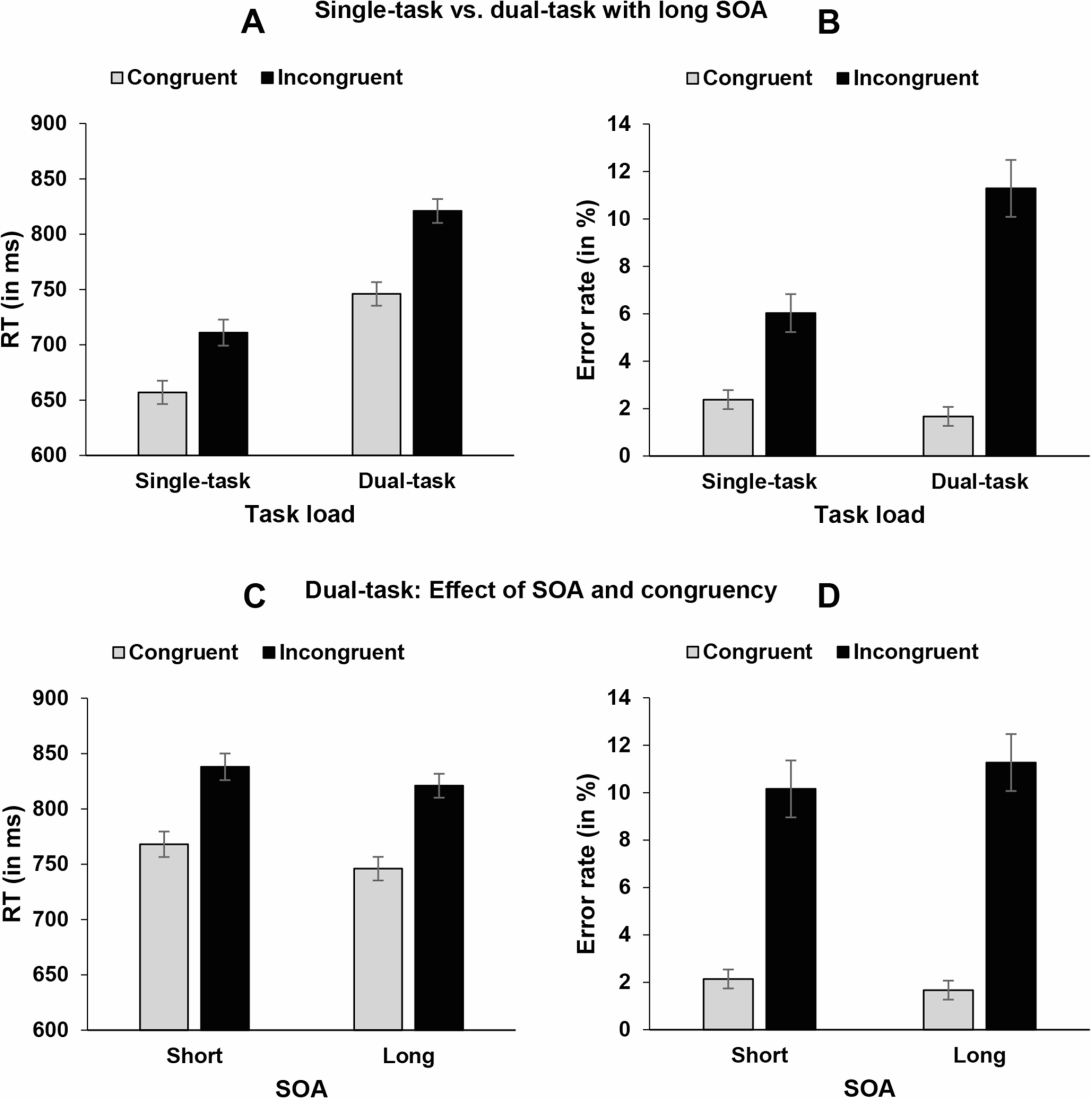
Panel A and B: Mean RT (in ms) and mean error rates (in %) of the spatial Stroop task as a function of task load (single-task vs. dual-task conditions with long SOA) and congruency (congruent vs. incongruent). Panel C and D: Mean RT (in ms) and mean error rates (in %) in the dual-task conditions of the spatial Stroop task as a function of SOA (short vs. long) and congruency (congruent vs. incongruent). Error bars show standard errors

A 2 × 2 repeated-measures ANOVA with the independent variables task load and congruency was conducted for the dependent variables RT and error rates (see Fig. 1, Panel A and B). Results showed a significant main effect of task load for RT (*F*(1,71) = 117.74, *p* <.001, η_p_² = 0.624) and error rates (*F*(1,71) = 13.38, *p* <.001, η_p_² = 0.159), indicating increased RT and error rates for the dual-task condition with long SOA compared to single-task condition (783 ms vs. 684 ms; 6.5% vs. 4.2%). Further, the main effect of congruency was significant for RT (*F*(1,71) = 173.00, *p* <.001, η_p_² = 0.709) and error rates (*F*(1,71) = 85.27, *p* <.001, η_p_² = 0.546), showing increased RT and error rates in incongruent trials compared to congruent trials (766 ms vs. 702 ms; 8.7% vs. 2.0%). Furthermore, the interaction between task load and congruency was significant for RT (*F*(1,71) = 8.55, *p* =.005, η_p_² = 0.108) and error rates (*F*(1,71) = 18.58, *p* <.001, η_p_² = 0.207), indicating a larger congruency effect in the dual-task condition with long SOA compared to the single-task condition (75 ms vs. 54 ms; 9.6% vs. 3.6%).

#### Dual-task condition: Effect of SOA and congruency

Moreover, for the dual-task conditions, a 2 × 2 repeated-measures ANOVA with the independent variables SOA and congruency was conducted for RT and error rates (see Fig. 1, Panel C and D). Results showed a significant main effect of SOA for RT (*F*(1,71) = 6.24, *p* =.015, η_p_² = 0.081), indicating longer RT with short SOA (803 ms) compared to long SOA (784 ms). This effect was not significant for error rates (*F* < 1; short SOA 6.2%; long SOA 6.5%). Further, the main effect of congruency was significant for RT (*F*(1,71) = 162.48, *p* <.001, η_p_² = 0.696) and error rates (*F*(1,71) = 68.36, *p* <.001, η_p_² = 0.491), showing increased RT and error rates in incongruent trials compared to congruent trials (829 ms vs. 757 ms; 10.7% vs. 1.9%). The interaction between SOA and congruency was neither significant for RT (*F* < 1; congruency effect with short SOA 70 ms; congruency effect with long SOA 75 ms) nor error rates (*F*(1,71) = 2.27, *p* =.136; congruency effect with short SOA 8.1%; congruency effect with long SOA 9.6%).

### Memory task (Task 2)

#### Single-task condition vs. dual-task condition with long SOA

The main focus of our study was on dual-task interference in long-term memory performance. As a first test, we ran a *t*-test with the independent variable task load, which showed a significant effect (*t*(71) = 5.77, *p* <.001, *d*_*z*_ = 0.680). Recall performance was worse in the dual-task condition with long SOA (27.5%) compared to the single-task condition (33.3%), reflecting dual-task costs in memory recall.

#### Dual-task condition: Effect of SOA and congruency

**Fig. 2:**
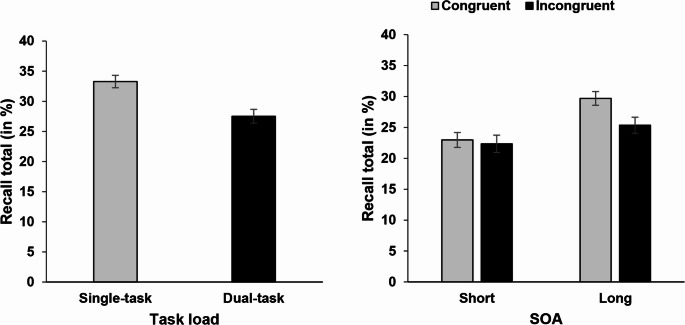
Left side: Mean recall total (in %) of the memory task as a function of task load (single-task vs. dual-task with long SOA). Right side: Mean recall total (in %) of the memory task as a function of SOA (short vs. long) and congruency in the spatial Stroop task (congruent vs. incongruent). Error bars show standard errors

Second, to test the effect of temporal task overlap and the influence of the processing conflict in the RT task on memory performance, we ran a 2 × 2 repeated-measures ANOVA with the independent variables SOA and congruency (see Fig. 2). This revealed a significant main effect of SOA (*F*(1,71) = 29.36, *p* <.001, η_p_² = 0.293), indicating reduced recall total for the short SOA (22.7%) compared to the long SOA (27.5%). Moreover, the main effect of congruency was significant (*F*(1,71) = 5.00, *p* =.028, η_p_² = 0.066), showing reduced recall total for words encoded in trials with incongruent Stroop stimuli (23.8%) compared to congruent stimuli (26.3%) in Task 1.

The interaction of SOA and congruency was non-significant (*F*(1,71) = 3.86, *p* =.053). Descriptively, the congruency effect was larger with long SOA compared to short SOA (−4,4% vs. −0.6%), but due to the statistical non-significance of this interaction effect, we refrain from drawing any conclusions, particularly given that statistical power was adequate for detecting effects of medium to large size. Hence, any interaction effect must have been quite small, if present at all.

## General discussion

The present study focused on dual-task interference in memory performance and investigated the impact of visual-manual response selection on auditory-verbal memory encoding in a PRP-like dual-task paradigm. To this end, participants were asked to auditorily encode words in either single-task or dual-task conditions, which needed to be verbally recalled after a short distractor activity. In the dual-task conditions, participants were presented with a visual stimulus from a spatial Stroop task (Task 1) that was only briefly presented on the screen. After either a short or long SOA, the auditory memory item (Task 2) was presented.

Results showed that recall performance in the memory task (Task 2) was worse in dual-task conditions with a long SOA compared to single-task conditions, reflecting global dual-task costs. Moreover, in the dual-task conditions, we found worse recall performance with short SOA compared to long SOA (i.e., a specific PRP-like effect of temporal task overlap), and most importantly, worse recall performance of memory items encoded during incongruent trials of the spatial Stroop task compared to congruent trials.

For the spatial Stroop task (Task 1), we found a standard congruency effect, which showed that the manipulation of congruency was successful. Compared to single-task conditions, the congruency effect was larger in dual-task conditions. Further, we found generally increased RT and error rates in dual-task conditions with a long SOA compared to single-task conditions, and in dual-task conditions we also found longer RT with short SOA compared to long SOA.

### Dual-task costs in memory encoding

The observed dual-task costs at the encoding stage of the memory task are in line with findings from previous research (e.g., Baddeley et al., 1984; Craik et al., 2018; Craik et al., 1996; Fernandes & Moscovitch, 2000; Hensen et al., 2024; Naveh-Benjamin et al., 2000). We assume that response selection in the RT task interferes with the process of encoding auditory-verbal information into memory at the “central” stage (Jolicoeur 1999a, b; Jolicoeur and Dell’Acqua 1998; Koch and Jolicoeur 2007; Koch et al. 2003; Koch and Prinz 2002, 2005). More specifically, the data suggests that response selection in Task 1 resulted in interference, possibly due to a delay in memory encoding that led to a decay of unconsolidated information and thus, to an information loss in Task 2 (see also Hensen et al., 2024). Although we used a long-term memory test to assess recall performance, we still believe that the reason for the observed dual-task costs is due to interference at the early stage of encoding information into memory, as it was shown in previous research that these early memory stages lay the ground for long-term memory formation (see e.g., Bartsch et al., 2024; Hartshorne & Makovski, 2019).

### Dual-task costs in response selection

For the spatial Stroop task, we also observed dual-task costs. This finding is in line with previous research (see e.g., Craik et al., 2018; Craik et al., 1996; Hensen et al., 2024). Because we found dual-task costs in both the memory task and RT task, one possible explanation could be that processes in these tasks interfere with each other at the capacity limited “central” stage due to capacity sharing (see e.g., CSS model; Tombu & Jolicoeur, 2003). Capacity sharing would not only lead to a decline in recall performance in the memory task, but also to impaired performance in the RT task, hence to mutual interference.

### Dual-task specific effects: The impact of SOA and congruency

**Fig. 3:**
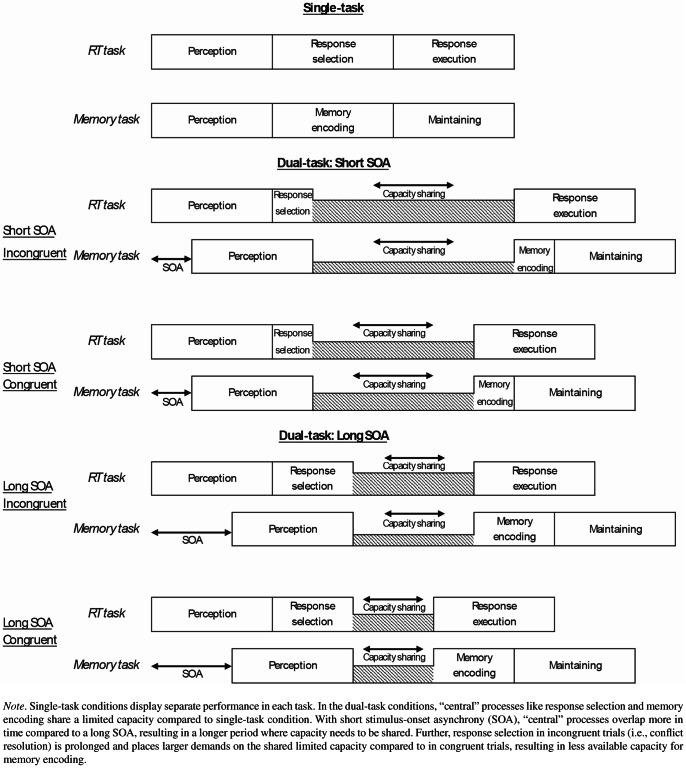
Processing in a RT task (Task 1) and memory task (Task 2) in a single-task condition and PRP-like dual-task conditions with short vs. long SOA and congruent vs. incongruent trials, based on assumptions of the capacity-sharing view

Focusing on the different dual-task conditions, the PRP-like SOA effect that we found in the memory task, combined with the increased RT with short compared to long SOA in the RT task, further support the idea of capacity sharing at the “central” stage and is in line with previous research using short-term memory tasks (e.g., Jolicoeur 1999a). Figure 3 depicts the possible mechanisms from a central capacity sharing view.

With short SOA, memory encoding and response selection have an overall longer phase of temporal overlap compared to with long SOA, resulting in a prolonged time in which “central” capacity needs to be shared between both tasks (e.g., Tombu & Jolicoeur, 2003). Consequently, the longer “central” capacity is shared, the less capacity is available for the individual tasks, and the more dual-task interference is observed in both tasks.

Besides the significant effect of SOA, we also found a significant impact of Stroop congruency on recall performance in the memory task. Memory items that were encoded during an incongruent trial of the RT task were less likely to be recalled compared to items encoded during a congruent trial. As incongruent trials are associated with a processing conflict at target identification and response selection (see Botvinick et al., 2001), we assume that conflict resolution might lead to an attentional prioritization of Task 1 processing over Task 2 processing. Hence, it could be that due to this prioritization, participants flexibly allocate a greater share of the shared limited capacity to processing demands of Task 1, resulting in less available capacity for processing demands in Task 2 in incongruent trials compared to congruent trials (see Fig. 3). Less available capacity for Task 2 processing would again lead to a possible delay in memory encoding and thus, a decay of unconsolidated information. This result would also be in line with research showing that memory performance is enhanced for conflict trials when the conflict is embedded within the memory item itself, due to selective attention demands (e.g., Rosner et al., 2015).

In a previous study (Hensen et al., 2024, Experiment 2), we investigated the dual-task effect of processing conflicts in a spatial Stroop task on recall performance in a free recall memory task, but there we could not find a clear effect. A key difference between the studies was the onset and duration of the stimulus presentation. In the previous study, stimulus presentation of both tasks was time-locked and the Stroop stimulus was presented for a long duration. In contrast, the present study used a PRP-like design with a strictly defined Task 1 (RT task) and Task 2 (memory task), and the Stroop stimuli were only briefly presented (see also Jolicoeur 1999a). These changes made it more likely that the Stroop stimuli were processed first in each trial and that response selection in the Stroop task was not postponed in time. Given these changes, we found in the present study that memory performance was indeed sensitive for the congruency manipulation in the Stroop task.

Taken together, the observed dual-task costs in the memory task and spatial Stroop task as well as the worse performance with short SOA compared to long SOA and incongruent trials compared to congruent trials can all be explained with some version of a flexible capacity sharing account. Yet, we note that one observation that would speak against such an account is the non-significant trend that showed that the congruency effect in the memory task was numerically more pronounced with long SOA compared to short SOA. Theoretically, in terms of capacity sharing, one would expect a larger congruency effect in memory performance and spatial Stroop performance with short SOA compared to long SOA. This is because the combination of short SOA and incongruent trials should place the largest demands on the shared limited capacity, and “central” processes should have the largest temporal overlap (see Fig. 3). However, we did not observe this interaction between SOA and congruency in both tasks and numerically, the memory task showed a reversed pattern of results. To avoid overinterpreting any non-significant interaction or trend, further research is needed to clarify whether there might be a relationship between SOA and congruency by, for example, introducing more fine-grained SOA variations.

Considering this unclear interaction between SOA and congruency, it is important to discuss other possible accounts for the observed dual-task interference between memory encoding and response selection. For example, as both the free-recall memory task and the spatial Stroop task relied on verbal processing, it could be that mutual dual-task interference is observed due to overlapping central codes. According to the multiple resource theory (Wickens, 2008), the concept of central codes refers to whether a task relies on verbal or spatial processing and the degree of interference between two tasks depends on the extent to which they draw on the same central codes. Consequently, two tasks that both require verbal processing should interfere more strongly with each other compared to tasks that depend on different central codes (e.g., verbal and spatial). Yet, in our previous study (Hensen et al., 2024, Experiment 1) we investigated the impact of overlap of central codes on dual-task interference between memory encoding and response selection. To manipulate the central code, in the spatial Stroop task we either visually presented location words (like in the present study) or symbolic arrows as stimulus material, but we did not find any significant impact of overlap of central codes on recall performance in the memory task. Thus, we assume that even if the stimulus material used in the spatial Stroop task involves verbal processing, the main task is still to classify the spatial meaning of the location words (i.e., the task relies more on spatial processing). In sum, we believe that it is unlikely that overlap of central verbal codes specifically contributed to the observed dual-task effects in the present study.

Another possible source of interference could be impaired advanced preparation for task-specific processes (see e.g., De Jong, 1995; De Jong & Sweet, 1994; see also Lyphout-Spitz et al., 2024). For example, De Jong and Sweet (1994) proposed that not only capacity limitations for “central” processes can account for dual-task costs, but also preparatory limitations. If two tasks require advanced preparation with a short temporal overlap between stimulus presentation, both Task 1 and Task 2 performance could suffer due to a less effective preparation. In contrast, with more time between stimulus presentations, more time for advanced preparation is available for both tasks. Thus, preparatory limitations could also explain the worse performance with short SOA compared to long SOA in the memory task and spatial Stroop task in the present study. Moreover, less effective preparation could also impair conflict resolution in the spatial Stroop task, resulting in worse performance in both tasks in incongruent compared to congruent trials. As the memory task and spatial Stroop task were not prepared in advance in the current study, we cannot rule out a contribution of preparatory limitations, so that future research is needed to further investigate the role of capacity limitations and preparatory limitations in auditory-verbal memory encoding by controlling the preparation time in both tasks.

Overall, the present study showed a PRP-like SOA effect in auditory-verbal memory encoding in a memory task with delayed free recall. As most previous research focused on visual memory tasks and immediate memory test (e.g., Jolicoeur 1999a), this is a novel contribution. Furthermore, and most importantly, we showed that a processing conflict in a visual-manual RT task modulates the success of auditory-verbal memory encoding, which further clarifies the underlying cognitive mechanisms of dual-task interference in terms of which processes interfere with each other.

## Conclusion

We investigated the impact of visual-manual response selection in a spatial Stroop task (Task 1) on auditory-verbal memory encoding in a free recall memory task (Task 2) in a PRP-like dual-task experiment. We found impaired recall accuracy in the long-term memory task in dual-task conditions with long SOA compared to single-task conditions, indicating that memory encoding suffers under dual-task demands with visual-manual response selection. Moreover, in the different dual-task conditions, we found worse recall accuracy with short SOA compared to long SOA (i.e., PRP-like SOA effect). Most importantly, recall accuracy was also lower for memory items that were encoded during incongruent trials of the spatial Stroop task compared to congruent trials. Together with the findings of worse performance in the spatial Stroop task under dual-task demands, results of the present study are mostly consistent with the idea of a flexible, limited capacity shared between response selection and memory encoding. However, since a capacity sharing view would also assume worse performance in incongruent trials at short SOA compared to long SOA in both tasks, which we did not observe, alternative explanations like overlapping central processing codes and preparatory limitations remain to be explored.

## Figures and Tables

**Table 1:**
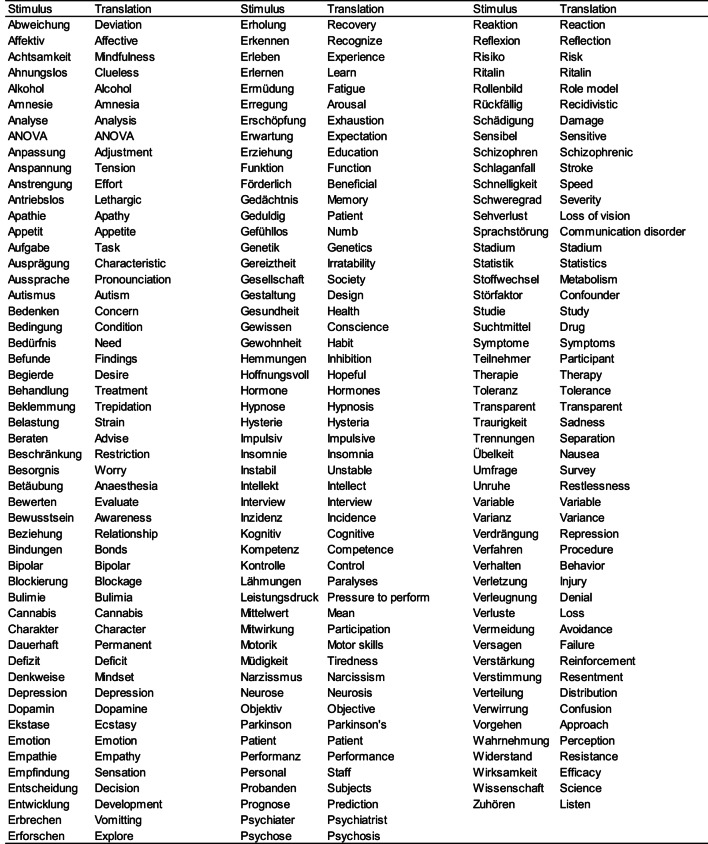
Full list of the 154 German psychology-related three-syllable words with English translations

## Data Availability

The datasets generated and analyzed during the current study are available in the OSF repository (https://osf.io/s3eby/overview?view_only=4a4d7d7050144eb6acc682260bed14d0). Pre-registration protocol is available at (https:/osf.io/yfp3w).

## Footnotes

1 There is also a line of research showing that memory performance is enhanced for conflict trials due to selective attention demands (e.g., Rosner et al., 2015). However, in these paradigms the conflict is implemented in the memory item itself, whereas we are interested in how memory performance in Task 2 is influenced by an independent task (Task 1) that involves the conflict.

## Code Availability

The experimental codes and stimuli from the current study are available from the corresponding author on reasonable request.
